# Establishing and maintaining biobanks: experience of Rajiv Gandhi Centre for Biotechnology, (RGCB) Trivandrum

**DOI:** 10.1186/1753-6561-7-S5-O16

**Published:** 2013-08-30

**Authors:** M Radhakrishna Pillai

**Affiliations:** 1 Rajiv Gandhi Centre for Biotechnology, Trivandrum, India

## 

A biobank is a depository for biomaterials from a representative portion of a human population and acts as a vault with intricate detailed information pertaining to the individuals from whom biological materials have been collected. Biobanks can be classified into population biobanks, biobanks for molecular epidemiology and biobanks for disease biology.

RGCB has been involved in biobanking with respect to three large studies in India namely i)Molecular Epidemiology of HPV in India, ii) A Randomized 2 versus 3 dose HPV Vaccination Clinical trial and iii) HPV AHEAD- : Role of Human Papilloma Virus Infection and other co-factors in the aetiology of Head & Neck Cancer.

A cervical cytology biobank has been established at the centre which is an almost inexhaustible resource for fundamental and applied biological research. It helps to understand the natural history of HPV infection and HPV induced lesions and cancers, screening effectiveness, exploration of new biomarkers, surveillance of the short- and long-term effects of the introduction of HPV vaccination. However legal and ethical principles concerning personal integrity and data safety must be respected strictly and biobank based studies require approval of ethical review boards.

The HPV vaccination study involves 10 different sites & different collaborating institutes across the country and all samples are shipped to RGCB, Trivandrum, which acts as a Central storage facility/ biobank. Sample shipment was an important procedure involving communication between base laboratory and biobank, import of registration database, strict temperature control, tracking of shipment and ready storage freezers on arrival. The sample verification process involves a temperature logger, freeze control tubes and bar code verification. The amount of clinical data linked to the samples determinate the availability and biological value of the sample. Thus acquiring the background information of each sample and cataloguing the available information systematically and meticulously is a must.

The major problems faced in the management of cervical cytology biobanks have been reclassification of smears before entry into database, failure of temperature logger, bar codes slipping off and maintaining uninterrupted power supply.

Publicly funded biobanks aim to promote the development of new knowledge by giving the research community access to data and samples. The most efficient way to acquire these benefits is to first maximize the use of biobanks in research and, second, to maximize the dissemination of knowledge developed by the research projects that used the biobanks. However there are issues with regard to the use of such stored materials especially when the demand occurs from the private sector. The decision is finally made by a scientific management committee consisting of members of World Health Organization (WHO) and the international agency for research on cancer (IARC).

